# The role of oral microbiota in the development of oral mucositis in pediatric oncology patients treated with antineoplastic drugs: a systematic review

**DOI:** 10.1186/s12903-024-03938-y

**Published:** 2024-02-05

**Authors:** Pierfrancesco Filetici, Sofia Germana Gallottini, Andrea Corvaglia, Martina Amendolea, Roberta Sangiovanni, Fabrizio Nicoletti, Antonio D’Addona, Leonardo Dassatti

**Affiliations:** 1 Department of Head and Neck and Sensory Organs, Università Cattolica del Sacro Cuore, Rome, Italy; 2 Unit of Oral Surgery and Implantology, Fondazione Policlinico Universitario A. Gemelli IRCSS, Rome, Italy

**Keywords:** Pediatrics, Neoplasms, Chemotherapy, Oral mucositis, Microbiota

## Abstract

**Background:**

In the pediatric oncology population, oral mucositis as a consequence of chemotherapy is a highly prevalent complication which strongly affects both the quality of life and treatment possibilities of the patients. Still, the etiopathological mechanisms carrying to its development are not fully understood, although a possible role of oral dysbiosis has been previously investigated with unclear conclusions. The aim of this systematic review was to assess the available evidence on the role of microbiota in the development of oral mucositis.

**Methods:**

A systematic literature search was performed following PRISMA guidelines. Three electronic databases were searched up until April 2023 and a following manual search included the reference lists of the included studies and reviews. Studies reporting microbiological and clinical data of pediatric patients treated by antineoplastic drugs were included.

**Results:**

Thirteen studies met the inclusion criteria, reporting an average mucositis prevalence of 57,6%. Candida albicans infections were frequently observed in studies performing microbiological analysis on oral lesions, in contrast with the low rate detection of the Herpes simplex viruses. Bacterial species such as coagulase-negative Staphylococci and Streptococcus viridans were detected more frequently on lesion sites. Studies reporting a quantitative analysis of the general flora did not show comparable results. Risk of bias assessment among studies was generally considered high or very high.

**Conclusions:**

While the specific role of certain microbiological agents, such as Candida albicans, was frequently reported among studies, data regarding the general dynamics of oral microbiota in the development of oral mucositis are lacking in the current literature. Thus, more studies are needed to provide the knowledge required in order to improve protocols for the prevention and treatment of this threatening complication.

## Introduction

Each year 429,000 children and adolescents aged 0 to 19 years are expected to develop cancer [1]. Many of the cancers affecting children are also able to affect adults, such as acute lymphoblastic leukemia (ALL), by far the most prevalent, followed by central nervous system (CNS) tumors, lymphomas, and bone cancers such as osteosarcoma and Ewing sarcoma [2]. Other cancers mainly affecting children are Neuroblastoma, Wilms tumor, rhabdomyosarcoma, and retinoblastoma [3]. The management of neoplastic pathologies encompass the use of chemotherapy, radiotherapy, and surgery contingent to the tumor’s type and anatomical site. Most commonly used chemotherapeutic agents are: vincristine, prednisone, L-asparaginase, anthracycline (doxorubicin or daunorubicin), high dose methotrexate, 6-mercaptopurine, cyclophosphamide, cytarabine, etoposide and thioguanine [4–6].

Chemotherapy acts on poorly differentiated or high-metabolism cells, thus affecting not only cancer cells, but also normal body cells [7, 8]. Therefore, there are several side effects that may be divided into either immediate or late signs of chronic toxicity [9]. One of the most common side effects of pediatric cancer treatment, oral mucositis, can be related to the frequent excretion of chemotherapeutic drugs through saliva, thereby exposing the oral cavity to their inherent toxicity [10]. Oral mucositis consists of oral mucosal damage and inflammation described as a five-phase process: initiation, primary damage response, signaling and amplification, ulceration, and healing [11, 12]. The World Health Organization (WHO), has introduced a system that grades oral mucosal lesions, with a 0 to 4 scale, based on clinical parameters: grade 0, no change; grade 1, soreness/erythema; grade 2, erythema, ulcers, can eat solid diet; grade 3, ulcers, can eat liquid diet only; grade 4, oral alimentation not possible [13, 14]. The incidence rate of oral mucositis ranges from 52 to 100% when young patients are submitted to standard chemotherapeutic protocols, however it becomes 100% when they receive highly dosed chemotherapy [15]. Other studies investigating the incidence of oral mucositis in young patients receiving chemotherapy show a range from 40 to 76% of cases [16].

To this day, there are still no guidelines indicating a standardized prophylaxis or treatment protocol for pediatric patients suffering from oral mucositis. Therefore, it is at the discretion of the oncologist, pediatrician, and dentist to determine the most appropriate treatment for the patient. Oral hygiene is associated with a lower incidence of oral mucositis [15], while cryotherapy and probiotics can reduce oral toxicity [17]. Soft laser treatments may be indicated in cases of ulcerative and refractory mucositis [17]. Additionally, the following pharmacological treatments could also be used: antioxidants, cytoprotective agents, cytokine production inhibitors and natural agents [18]. Local mouthwashes with 0.2% chlorhexidine and morphine may even allow better pain control compared to systemic analgesic treatment [19].

The etiology of oral mucositis is poorly known. A pathophysiologic process has been hypothesized, consisting of a cascade of biological and immunological events, causing cell apoptosis and damaging connective tissue, followed by a series of signaling inflammatory pathways sparked by the presence of cytotoxic chemicals and worsened by systemic and local factors [20–22]. Specifically, systemic factors include chemotherapeutic drugs. Important variables influencing the severity of mucosal injury include the type of chemotherapy medicines used, their dosage, and the schedule of administration [22, 23]. Among the drugs most often associated with mucositis there are: alkylating agents, anthracyclines, platinum compounds, antimetabolite agents, antibiotics, vinca alkaloids and taxanes [18, 24, 25].

Systemic antineoplastic treatments may induce alterations in patients’ oral microbiome, and dysbiosis may be implicated in the onset of oral mucositis [26]. The antimicrobial effects of chemotherapy favor the dominance of gram-negative anaerobes over oral streptococci [27]. Gram-negative bacteria can worsen or accelerate the development of ulcers by releasing endotoxins called lipopolysaccharides (LPS), which induce macrophages to produce inflammatory molecules such as TNF-, IL-6, and IL-1 [22]. This chain of events can ultimately culminate in the manifestation of oral mucositis [22]. However, the specific role of bacterial species in the development of ulcerative mucositis remains unknown [28].

To the best of authors knowledge, only a few studies have investigated the role of the oral flora in the development of oral mucositis in patients undergoing chemotherapy, especially in the pediatric population, and a scarcity of systematic reviews is noticeable. Given the clinical importance of the treated topic and the growing interest and requested knowledge of microbiota-related conditions, the goal of this systematic review was to assess the role of oral dysbiosis associated with antineoplastic drugs in the development of oral mucositis in young oncology patients (< 18 years old).

## Materials and methods

### Research strategy

The PRISMA (Preferred Reporting Items for Systematic Reviews and Meta-Analyses) guidelines were used to develop the protocol for this research. The Patient-Intervention-Comparison-Outcome (PICO) question was employed to formulate a clear study aim: Patients under 18 years of age, of any gender and ethnicity, at diagnosis of any type of childhood cancer (P = Patient); oral health assessment at the time of neoplasm diagnosis before and during chemotherapy (I = intervention); not considered (C = Comparison); assessment of quantitative and qualitative change in the oral microbiota; evaluation of oral mucositis’s incidence (O = outcome).

### Selection process

A bibliographic search was conducted upon three different scientific digital databases, namely MEDLINE, WOS and SCOPUS. For this research, the following algorithm was established: (child OR pediatrics OR pediatric OR infant) AND (neoplasms OR neoplasia OR neoplasias OR tumor OR malignancy OR malignancies OR cancer) (stomatitis OR oromucositis OR oromucositides OR mucositis) AND (chemotherapy OR antineoplastic treatments).

### Eligibility criteria

All original articles, case reports and case series, with no restrictions regarding year of publication and type of study meeting the following inclusion criteria were included:

- studies reporting data regarding patients under 18 years of age with diagnosis of any type of childhood cancer undergoing chemotherapy as an antineoplastic therapy protocol evaluated from baseline;
- studies reporting clinical and microbiological data upon the development of oral mucositis as a treatment complication.

Exclusion criteria were defined as follows:

- studies including patients undergoing further therapies, such as radiotherapy;
- studies that did not perform an oral evaluation at baseline;
- studies including patients with other systemic pathologies or comorbidities.

### Data collection

The team conducted study selection by reviewing the titles and abstracts obtained by the digital research and only those that met the eligibility requirements were evaluated. The remaining articles were then thoroughly read, and the unselected articles were discarded. Abstracts of reviews obtained by the digital research, described above, were also examined. From those included in the first step selection, citations of articles related to the topic were extracted. The abstracts of these articles were then subjected to the same analysis as all the other ones. Article selection process was always performed independently by two reviewers, and a third reviewer’s opinion was claimed if disagreement among the two reviewers occurred. Data extraction was performed on included articles in order to create a database with all the variables accessible on each article that were pertinent to the study. When available, the following details were taken from each chosen study: type of study, number of patients, age range of patients, number of test group, characteristics of test group, number of control group, characteristics of control group, type of antineoplastic therapy, mucositis diagnosis criteria, mucositis preventive intervention, mucositis treatment intervention, timing of clinical evaluation, timing of microbiological evaluation, type and site of microbiological evaluation, mucositis prevalence and microbiological evaluation results.

### Risk of bias assessment

A quality assessment of observational epidemiological studies was performed independently by two of the review authors by means of the ROBINS-E—Risk of Bias In Nonrandomized Studies—of Exposure (Higgins J. et al., 2022); the risk was defined as very high, high, generating some concerns and low. It is based on the analysis of 7 domains: (D1) risk of bias due to confounding factors, (D2) risk of bias from exposure measurement, (D3) risk of bias in selection of study participants (or analyses), (D4) risk of bias due to postexposure interventions, (D5) risk of bias due to missing data, (D6) risk of bias from outcome measurements, and (D7) risk of bias in selection of reported outcomes. The risk was defined as follows:

(A) “low”: domain 1 (D1) was judged as low risk or with some concern, and if all other domains were considered low risk;
(B) “with some concern”: at least one domain was at some concern but no domain was at high or very high risk of bias;
(C) “high”: at least one domain was at high risk of bias but no domain was at very high risk of bias OR if several domains generated some concern;
(D) “very high”: at least one domain is at very high risk OR if several domains are at high risk of bias.

Case series were differently analyzed by means of specific quality assessment tools developed by the National Institutes of Health (NIH) in 2013.

The quality of each study was rated as “good,” “sufficient,” or “poor” after answering the following 9 questions: a) was the study question or objective clearly stated?; b) was the study population clearly and fully described, including a case definition?; (c) were the cases consecutive?; (d) were the subjects comparable?; (e) was the intervention clearly described?; (f) were the outcome measurements clearly defined, valid, reliable, and implemented consistently across all study participants?; (g) was the length of follow-up adequate?; (h) were the statistical methods well described?; (i) were the results well described? The possible answers to the questions were: yes, no, cannot determine/not applicable/not reported.

Case reports were evaluated using the critical appraisal checklist (JBI critical appraisal checklist, 2017 version) developed by the Joanna Briggs Institute. The overall assessment led to “inclusion” or “exclusion” of a study in the systematic review or even the possible “need to seek further information” to make a judgment. Judgment is made after answering the following 8 questions: 1. Were the patient’s demographic characteristics clearly described?, 2. Was the patient’s history clearly described and presented as a timeline?, 3. Was the patient’s current clinical condition clearly described? 4. Have the diagnostic tests or assessment methods and results been clearly described?, 5. Have the interventions or treatment procedures been clearly described?, 6. Has the postintervention clinical condition been clearly described?, 7. have adverse events (harms) or unexpected events been identified and described?, 8. does the case report provide take-home messages? The 4 possible response options were “yes”, “no”, “unclear”, “not applicable”.

Other possible sources of bias were not considered.

### Data analysis

The overall mean prevalence of mucositis was calculated as weighted mean of prevalence values of mucositis expressed in each study. Whenever the prevalence assessment was performed at different times of therapy or through a clusterization of the study population, a mean value of prevalence values obtained from each study was considered.

Case series and case reports were excluded from the weighted mean calculation.

## Results

**Fig. 1:**
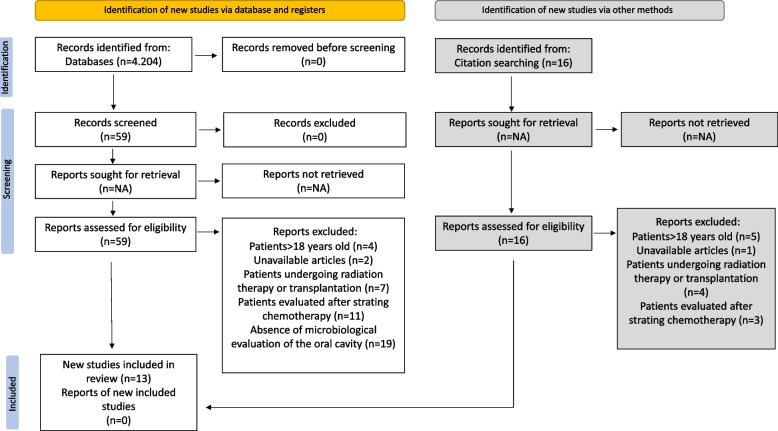
PRISMA diagram

The database search led to the identification of 4204 articles. Following the selection process outlined in the PRISMA diagram, 13 studies met the inclusion criteria, and their data were analyzed in this systematic review (Fig. 1) [29–41].

The included studies were published between 1998 and 2021. Among them, 9 were longitudinal studies (5 case–control studies, 4 cohort studies), 2 were retrospective studies, and 2 were case reports/series (Table 1) [29–41].

**Table 1:** Articles included in the review

Article first author + year	Type of study	n Patients	Age range	n test group	Characteristics test group	n control group	Characteristics control group	Type of antineoplastic therapy
*Anirudhan 2008* [29]	Retrospective study	70	< 15 years	70	Patients with leukemia	-	-	MCP-841 protocol: Induction (Vincristine, Daunorubicin, L-Asparaginase, Prednisolone, Methotrexate); Induction 2 (6-Mercaptopurine, Cyclophosphamide, Methotrexate); Consolidation (Cyclophosphamide, Vincristine, Cytarabine, 6-Mercaptopurine, Prednisone); Maintenance (Vincristine, Daunorubicin, L-Asparaginase, Methotrexate, 6-Mercaptopurine)
*Bardellini 2017* [30]	Case report	1	16 years	1	Stage 3 Burkitt’s lymphoma	-	-	Chemotherapy (5 days combination of Vincristine, Methotrexate, Ifosfamide, Cytarabine and Etoposide)
*Costa 2020* [31]	Cohort study	26	4–18 years	26	Patients that have not started the chemotherapy at base-line period	-	-	Chemotherapy (not specified protocols)
*De Oliveira 2019* [32]	Case series study	9	2–14 years		Diagnosis of ALL	-	-	GBTLI ALL 2009 protocol: combination of Dexametasone, Vincristin, Daunomycin, intrathecal chemotherapy (methotrexate, cytarabine and dexamethasone), L-Asparaginase, 6-Mercaptopurine, Cytarabine, Leucovorin, Methotrexate
*Gandhi 2017* [33]	Cohort study	62	1–14 years		Pediatric oncological patients	-	-	Chemotherapy (not specified protocols)
*Juarez-Lopez 2018* [34]	Case control study	103	4–15 years	73	Chemotherapy	30	No chemotherapy	Chemotherapy (Methotrexate, Vinblastine e Vincristine, Bleomycin)
*Levy-Polack 1998* [35]	Case control study	96	1–16 years	36	Preventive protocol of mucositis	60	No preventive protocol of mucositis	Chemotherapy (not specified protocols)
*Mendonca 2012* [36]	Cohort study	71	3–276 months	71	Diagnosis of pediatric ALL	-	-	Induction phase with MADIT (prednisone, vincristine, doxorubicin, L-asparaginase, Methotrexate, ara-C and dexamethasone). Consolidation phase with cyclophosphamide, cytarabine and 6-mercaptopurine. Intensification phase with methotrexate, 6-mercaptopurine and MADIT
*Olczak-Kowalczyk 2012* [37]	Case control study	45	1,5–18 years	20(A) + 14(B)	Organ recipients (A) + antitumor chemotherapy (B)	11	Group without secondary immunodeficiency	Group A immunosoppressive treatment (steroids, cyclosporine, tacrolimus, sirolimus). Group B followed protocols for each tumor: MB protocol (isophosphosphamide, eto- poside, cisplatin, vincristine, carboplatin); COPADM protocol (cyclophosphamide, vincristine, prednisone, adriamycin, methotrexate); SIOPEL protocol (cisplatin, doxorubicin)
*Pinto 2018* [38]	Retrospective trasversal study	71	1–16 years	71	Patients with ALL undergoing chemotherapy	-	-	Chemotherapy (not specified protocols)
*Sixou 1998* [39]	Case control study	32	3–16 years	16	Patients with cancer undergoing first treatment	16	Absence of general and oral pathology	FRALLE 93 Protocol: combination of Prednisolone, Daunorubicin, Vincristine, L-Asparaginase, Methotrexate, Cytarabine, Hydrocortisone, Etoposide, 6-Thioguanine, 6-Mercaptopurine, Doxorubicin, Dexemethasone, Vindesine LAME 91 Protocol: combination of Cytarabine, Mitoxantrone, Etoposide, Daunorubicin, Asparaginase, Amsacrine, intrathecal Cytarabine, Methotrexate and steroid, 6-mercaptopurine) LMB 89 Protocol: combination of Vincristine, Cyclophosphamide, Prednisone, Methotrexate, Adriamycin
*Soares 2011* [40]	Cohort study	17	2–12 years	-	Patients with cancer undergoing chemotherapy	-	-	Chemotherapy (not specified protocols)
*Ye 2013* [41]	Case control study	75	4–18 years	37	Pediatric oncological patients	38	Pediatric healty population	Chemotherapy (not specified protocols)

The included articles analyzed in the systematic review underwent risk of bias assessment using the ROBINS-E (Risk of Bias In Nonrandomized Studies—of Exposure) tool. All the studies were found to have a high or very high risk of bias (Table 2).

**Table 2:**
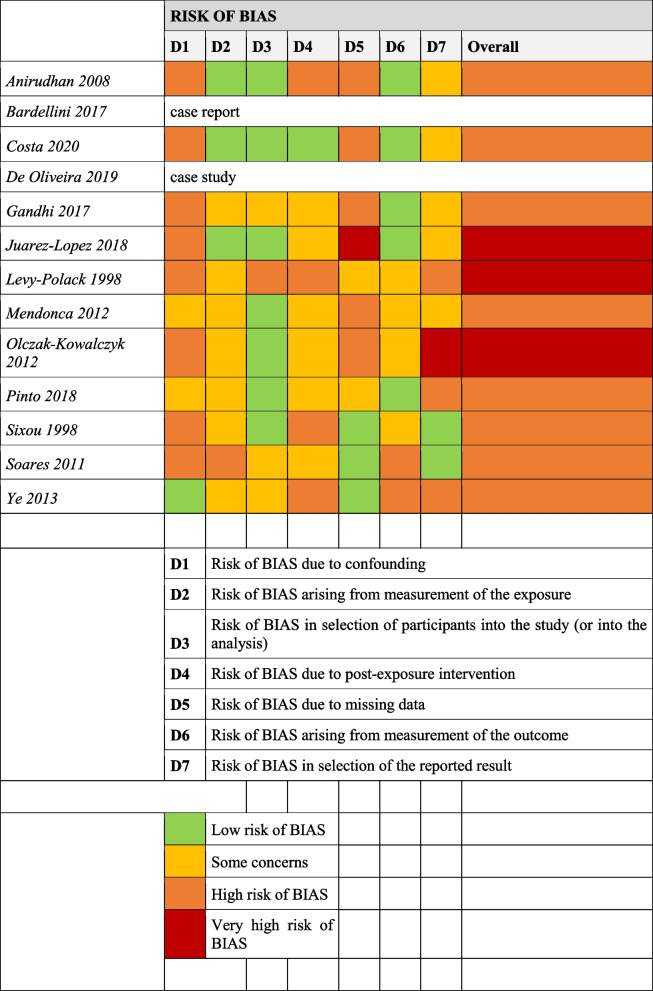
Risk of bias assessment [29–41]

The case series by De Oliveira et al. (2019) was analyzed and included in the review through a specific quality assessment developed by the National Institutes of Health (NIH) in 2013 (Table 3) [32]. The study was found to be of good quality.

**Table 3:** Quality assessment tool for case series

The National Institutes of Health (NIH) quality assessment tool for case-series study (Interventional)			
*De Oliveira 2019* [32]			
**Major Components**	**Response options**		
1. Was the study question or objective clearly stated?	**Yes**	No	Cannot Determine/ Not Applicable/ Not Reported
2. Was the study population clearly and fully described, including a case definition?	**Yes**	No	Cannot Determine/ Not Applicable/ Not Reported
3. Were the cases consecutive?	Yes	**No**	Cannot Determine/ Not Applicable/ Not Reported
4. Were the subjects comparable?	Yes	**No**	Cannot Determine/ Not Applicable/ Not Reported
5. Was the intervention clearly described?	**Yes**	No	Cannot Determine/ Not Applicable/ Not Reported
6. Were the outcome measures clearly defined, valid, reliable, and implemented consistently across all study participants?	**Yes**	No	Cannot Determine/ Not Applicable/ Not Reported
7. Was the length of follow-up adequate?	**Yes**	No	Cannot Determine/ Not Applicable/ Not Reported
8. Were the statistical methods well-described?	Yes	**No**	Cannot Determine/ Not Applicable/ Not Reported
9. Were the results well-described?	**Yes**	No	Cannot Determine/ Not Applicable/ Not Reported
**Quality Rating**	**GOOD**	FAIR	POOR
Additional Comments (If Poor, please state why):			

The case report by Bardellini et al. (2017) was evaluated using the JBI critical appraisal checklist (2017 version) developed by the Joanna Briggs Institute (Table 4) [30]. The study was included because it meets 5 out of the 8 critical appraisal checklist components that, according to JBI, a proper study should have.

**Table 4:** Critical appraisal checklist for case reports

The Joanna Briggs Institute (JBI) Critical Appraisal Checklist for Case Reports (last amended in 2017)				
*Bardellini 2017* [30]				
**Major Components**	**Response options**			
1. Were patient’s demographic characteristics clearly described?	Yes	**No**	Unclear	Not applicable
2. Was the patient’s history clearly described and presented as a timeline?	Yes	**No**	Unclear	Not applicable
3. Was the current clinical condition of the patient on presentation clearly described?	**Yes**	No	Unclear	Not applicable
4. Were diagnostic tests or assessment methods and the results clearly described?	**Yes**	No	Unclear	Not applicable
5. Was the intervention(s) or treatment procedure(s) clearly described?	**Yes**	No	Unclear	Not applicable
6. Was the post-intervention clinical condition clearly described?	**Yes**	No	Unclear	Not applicable
7. Were adverse events (harms) or unanticipated events identified and described?	Yes	**No**	Unclear	Not applicable
8. Does the case report provide takeaway lessons?	**Yes**	No	Unclear	Not applicable
**Overall appraisal:**	**Include**	Exclude	Seek further info	

For each study, the diagnosis criteria of mucositis, prophylaxis and treatment of mucositis, evaluation timing of lesions in relation to chemotherapy administration, and prevalence were highlighted (Table 5). The overall weighted mean frequency of mucositis was found to be 57,6%.

**Table 5:** Mucositis analysis for each study

**Article first author + year**	**n patients**	**Mucositis diagnosis criteria**	**Mucositis preventive intervention**	**Mucositis treatment intervention**	**Timing of clinical evaluation**	**Mucositis prevalence**	%
*Anirudhan 2008* [29]	70	WHO criteria	antibiotics/steroids usage in previous two weeks	Initial treatment included topical analgesics, oral antifungals or acyclovir. Amphotericin B was started if persisted beyond 5 days of fluconazole or antibiotics.	T1: chemotherapy induction T2: chemotherapy intensification T3: 18 months of maintenance phase	T1: 41 episodes T2: 39 episodes T3: 20 episodes	47,6%
*Bardellini 2017* [30]	1	WHO criteria	Twice weekly Cotrimoxazole prophylaxis	Antibiotic combination therapy with Amikacin and Ceftazidime for 8 days	T1: Before initiation of cancer therapy T2: During II cycle of cancer therapy T2b Phase II (consolidation); T2c Phase III (delayed intensification); T2d Phase IV (maintenance) T3: Post-treatment (disease-free) T4: Relapse cases Tx: Un-sp	Not specified	
*Costa 2020* [31]	26	Modified OAG	Not specified	Not specified	T0: before cancer therapy T1: after 2 weeks of treatment T2: after 5 weeks of treatment T3: after 10 weeks of treatment	T1: n = 4 cases T2: n = 7 cases T3: n = 5 cases	20,5%
*De Oliveira 2019* [32]	9	WHO criteria	Not specified	Not specified	D0/1, D8, D15, D35 of pre-phase and induction phase; D1, D15, D29, D50 of consolidation phase	0 cases	
*Gandhi 2017* [33]	62	WHO criteria	Not specified	Not specified	First phases of chemotherapy	58.1%	58,1%
*Juarez-Lopez 2018* [34]	103	Clinical evaluation	Not specified	Not specified	Not specified	98% at starting of chemotherapy. Induction phase stricly related with mucositis incidence (OR = 7,6, IC 95% p = 0,0001)	98%
*Levy-Polack 1998* [35]	96	NCI Grading scale	Mouthwash with sodium bicarbonate and water after every meal; a mouthwash with a nonalcoholic solution of chlorhexidine (0.12%) twice a day (midmorning and evening); cleaning of mucosa with gauze soaked in iodopovidone, four times a day prior to the use of nystatin. The children “swishing and swallowing” with nystatin 500,000 units, oral suspension four times a day and a daily rinse with sodium fluoride 0.05% (nonalcoholic solution).	Not specified	T1: diagnosis; T2: chemotherapy starting T3: evaluation at D7 and D14 of treatment	The overall difference between the two groups was not statistically significant but a decrease after the protocol application was found in the study group Group I: 30% Group II: 21.9%	26,05%
*Mendonca 2012* [36]	71	NCI Grading scale	Not specified	In some cases, use of Acyclovir	T1: beginning of treatment T2: D14 T3: D56	Day 14 (71 pz) n(%): GRADE 0: 6 (8.3) GRADE I: 47 (65.3) GRADE II: 10 (13.9) GRADEIII: 8 (11) Day 56 (67 pz) n(%) GRADE 0: 29 (43.3) GRADE I: 25 (37.4) GRADE II: 7 (10.5) GRADE III: 6 (8.8)	24,3%
*Olczak-Kowalczyk 2012* [37]	45	Common Terminology Criteria Adverse Events (CTCAE),	Not specified	Not specified	T1: patient enrollement	GROUP B: 14 patients had mucositis (its severity was assessed according to the CTCAE scale: I = 6, II = 3, III = 5)	31,1%
*Pinto 2018* [38]	71	Clinical evaluation	Chlorhexidine 0.12%	Lasertherapy and chlorhexidine	Not specified	44/71—72%	72%
*Sixou 1998* [39]	32	Clinical evaluation	Mouth washing three times a day with an alchool-free 2% chlorhexidine solution	-	D0 (before chemotherapy), D7, D14, D21 of treatment	8/16—50%	50%
*Soares 2011* [40]	17	Clinical evaluation	Mouth washes wiht 0.12% chlorhexidine gluconate solution twice a day, 30 min after breakfast and after the last meal for 10 days	-	Daily during the period of hospitalization of the patient for chemotherapy	5/17—29,4%	29,4%
*Ye 2013*	75	WHO criteria	Single 2.5 mg/mL benzydamine-based mouth rinse for the period of chemotherapy		Not specified	25/37	67,6%
					**Mean prevalence of mucositis:**		**57,6%**

Methodology of included studies has been summarized in Table 6.

**Table 6:** Methodology of included studies

Article first author + year	Microbiological methodology	Outcome
*Anirudhan *et al*. 2008* [29]	Culture and serology for bacteria, herpes simplex and fungi. Urine test for presence of fungal elements	Qualitative analysis Prevalence
*Bardellini *et al*. 2017* [30]	Culture not specified	Qualitative analysis Prevalence
*De Oliveira 2019* [32]	PCR analysis for detection of DNA of HSV-1, EBV and CMV. PCR for the β-globin constitutive gene to control false-positive results.	Qualitative analysis Prevalence
*Mendonca 2012* [36]	Primer PCR technique for HSV-1 detection PCR for the β-globin constitutive gene to control false-positive results. Blood, chocolate and McConkey agar for bacteria cultures; Sabouraud agar for fungi cultures.	Qualitative analysis Prevalence
*Olczak-Kowalczyk 2012* [37]	Culture on solid media: blood agar, chocolate agar and the Sabouraud agar. Bacteria identified upon Gram-staining. Biochemical identification using commercially available kits: GP and GN cards, the VITEK 2 system, PYR and the optochin sensitivity test. Density of yeast colonies was assessed according to a 4-tiered scale: 0 – no fungal growth; 1 – < 102 CFU ⁄ ml; 2 – 102 to 103 CFU ⁄ ml; 3 – over 103 CFU ⁄ ml. Yeast type was determined using the ID32 test	Qualitative analysis Prevalence Quantitative analysis (density of yeast colonies)
*Sixou 1998* [39]	Inoculation on non-selective medium and selective media: TSBV, TBBP, and hypersaline agar. Bacterial profiles obtained with API ZYM1, Rapid Id32A1 and Rapid Id Strep1 identification systems. Culture on Columbia blood agar enabled the determination of the total anaerobic viable count (TAVC).	Qualitative analysis Prevalence Quantitative analysis (TAVC)
*Soares 2011* [40]	Culture on solid media: mannitol salt agar, MacConkey agar, cetrimide agar, and Sabouraud agar. Characteristics determined by the Gram method modified by Kopeloff-Beerman. Gram-negative bacteria were identified by Mini-API identification system. Yeast-like fungi identified as Candida albicans by staining of the colonies on CHROMagar.	Qualitative analysis Prevalence
*Ye 2013* [41]	Bacterial samples analyzed using 454 FLX pyrosequencing with minor modifications. The PCR products were sequenced using a two-lane PicoTiterPlate on a Genome Sequencer FLX system. Denoised sequences were aligned and sorted into operational taxonomic units (OTUs).	Qualitative analysis Prevalence Quantitative analysis (Unifrac)
**Clinical methodology**		
*Gandhi *et al*. 2017* [33]	Evaluation of characteristic conditions such as white lesions (candidiasis) or vesicles and/or ulcers (HSV) with different symptoms as pain, burning and others. Systematical examination of buccal and sulcular mucosa, the tongue, the floor of the mouth, the hard and soft palate, the fauces, and free and attached gingiva	
*Juarez-Lopez *et al*. 2018* [34]	Interviewed family and/or caregivers of the participating children to investigate about present symptoms A dentist examined lips, lanes, palate, oropharynx and tongue	
*Levy-Polack *et al*. 1998* [35]	Diagnosis of oral candidiasis as white, adherent plaque on the oral mucosa or tongue that, if scraped, left a bleeding base	
*Pinto *et al*. 2018* [38]	Intraoral examination to identify abnormalities and oral lesions	

In order to better organize the information gained through data collection, the microbiological data from included studies were divided into two sections.

First section involved data collected from studies that performed a direct sampling from sites showing signs of oral mucosa lesion. The second section, conversely, involved data obtained from studies that sampled other areas of the oral cavity, thus reflecting the general oral microbiological profile of the patients rather than the specific microbiota associated with the lesion area.

### Direct sampling on oral lesions

Studies by Anirudhan (2008), Olczak-Kowalczyk (2012), and Bardellini (2017) reported microbiological data obtained through direct sampling of sites with lesions (Table 7) [29, 30, 37]. Coagulase-negative Staphylococci and Streptococcus viridans were found with a relative high percentage in both Olczak-Kowalczyk’s (2012) and Anirudhan’s (2008) studies [29, 37].

**Table 7:** Prevalence of infection by different bacterial and fungal species in oral lesions

Microbiological evaluation of oral lesions			
	*Anirudhan *et al*. 2008* [29]	*Olczak-Kowalczyk *et al*. 2012* [37]	*Bardellini *et al*. 2017* [30]
	70 patients	14 patients	1 patient (case report)
**Bacteria**			
*Staphylococcus aureus*	4 (14.2%)	1 (7.1%)	
*Coagulase negative Staphylococci*	3 (10.7%)	9 (64.2%)	
*Staphylococcus epidermidis*	1 (3.6%)		
*Streptococcus viridans*	4 (14.2%)	12 (85.7%)	
*Streptococcus bovis*		2 (14.2%)	
*Streptococcus salivarius*		1 (7.1%)	
*Group b Streptococcus*	1 (3.6%)		
*Leuconostoc spp.*		1 (7.1%)	
*Enterococcus spp.*	1 (3.6%)	1 (7.1%)	
*Neisseria spp.*		4 (28.5%)	
*Pseudomonas aeruginosa*	3 (10.7%)	0	
*Enterobacter cloacae*		1 (7.1%)	
*Klebsiella oxytoca*		1 (7.1%)	
*Klebsiella pneumoniae*	3 (10.7%)	1 (7.1%)	
*Haemophilus parainfluenzae*		1 (7.1%)	
*Haemophilus influenzae*			
*Prevotella melaninogenica*			
*Prevotella disiens*			
*Veillonella*	3 (10.7%)		
*Peptostreptococcus*	1 (3.6%)		
*Lactococcus*	1 (3.6%)		
*Raoultella planticola*			1 (100%)
**Fungi**			
*Trichosporon*	1 (2.6%)		
*Aspergillus*	4 (10.2%)		
*Rhodotorula*	1 (2.6%)		

Six studies reported data regarding clinical and/or microbiological detection of herpetic and/or fungal lesions from direct sampling of oral lesions [27, 33–35, 37, 38]. Among these, three detected Herpes simplex and Candida, while the others only detected Candida. The prevalence of Candida albicans was high (50–60%) in both the studies by Anirudhan (2008) and Olczak-Kowalczyk (2012), which performed microbiological testing on the specimens, with an overall percentage among the studies ranging from 16,1% to 78% (Table 8) [29, 37].

**Table 8:** Prevalence of Candida albicans and herspes simplex virus infections in oral lesions

**Study**	**Sample**	**Candida Prevalence**	**Method of assessment**
*Levy-Polack *et al*. 1998* [35]	96	23/96 (24%)	Clinical evaluation
*Anirudhan *et al*. 2008* [29]	70	38/70 (52.3%)	Microbiological evaluation of oral swabs
*Olczak-Kowalczyk *et al*. 2012* [37]	34	21/34 (61.7%)	Microbiological evaluation of oral swabs
*Gandhi *et al*. 2017* [33]	62	10/62 (16.1%)	Clinical evaluation
*Juarez-Lopez *et al*. 2018* [34]	73	57/73 (78%)	Clinical evaluation
*Pinto *et al*. 2018* [38]	71	9/71 (13.1%)	Clinical evaluation
**Study**	**Sample**	**HSV Prevalence**	**Method of assessment**
*Anirudhan *et al*. 2008* [29]	70	1/70 (3%)	Microbiological evaluation of oral swabs
*Gandhi *et al*. 2017* [33]	62	6/62 (9.7%)	Clinical evaluation
*Pinto *et al*. 2018* [38]	71	2 (3.3%)	Clinical evaluation

HSV infection was less frequent, with a range between 3 and 9,7% of the population.

### Sampling in standardized sites of the oral cavity

Studies by Sixou et al. (1998) and Ye et al. (2013) evaluated the variation in the complexity of the oral bacterial flora and the percentage variation of certain bacterial species during chemotherapy treatment [39, 41].

The study by Sixou et al. (1998) showed how healthy patients have a consistently greater complexity of microbial flora in comparison to oncology patients undergoing chemotherapy on days 0, 7, 14, and 21 of chemotherapy treatment [39].

Oncology patients, however, showed a non-significant microbiological variation during therapy.

Meanwhile, in the study by Ye et al. (2013), the UniFrac distance, calculated from the diagnosis of neoplasia to the end of chemotherapy treatment, was higher in patients who developed mucositis (> 0.4 UniFrac distance) compared to those in whom it did not occur (< 0.4 UniFrac distance) [41].

UniFrac measures the distance between microbial communities based on the phylogeny of the operational taxonomic unit (OTU) [42]. It considers the presence or absence of an OTU in a community, rather than its abundance.

Regarding the percentage variation of different bacterial species detected in the oral cavity, in the total count of viable anaerobes before and after chemotherapy both studies analyzed the abundance of Capnocytophaga spp.

In the study by Sixou, the percentage of Capnocytophaga spp. was higher in healthy patients than in sick patients, whilst in sick patients there was no significant variation of the percentage during chemotherapy treatment [39].

In the study by Ye, a higher concentration of Capnocytophaga spp. was observed at the time of neoplasia diagnosis in patients who developed mucositis after chemotherapy than in those who did not develop mucositis [41].

The comparison of the frequency of different microorganisms in patients undergoing chemotherapy, following sampling from non-lesion sites of the oral cavity, is described in Table 9. Coagulase-negative Staphylococci had frequency percentages ranging from 25 to 80%, regardless of mucositis, after starting chemotherapy. Higher percentages were observed in patients with mucositis. Candida albicans, regardless of mucositis, ranged from 15 to 60%, with higher percentages in patients with mucositis. HSV, according to Mendoca 2012, showed a decrease in percentage from Day 14 to Day 56 of antineoplastic treatment [36]. Viridans streptococci and Capnocytophaga showed alternating trends between Day 0 and Day 21 of chemotherapy, with an increase in Viridans streptococci and a decrease in Capnocytophaga at the end of the treatment.

**Table 9:** Frequency of microorganisms sampled in oral cavity

			**Microrganism’s frequency (%) in patients with mucositis**		**Microrganism’s frequency (%) in patients with no mucositis**			**Microrganism’s frequency (%) in patients with or without mucositis**				
**Microrganism**	**Article & Year**	**Sampling site**	D0	Dx	D0	D8	Dx	D0	D7	D14	D21	D56
**Coagulase-negative staphylococci**	*Soares 2011* [40]	Labial and buccal mucosa		80			33,3					
	*Ye 2013* [41]	Lower lip and on the bucca	0	48								
	*Sixou 1998* [39]	Supragingival plaque of the last three teeth of the upper right quadrant and the last three teeth of the lower left						25	25	25	25	
**Klebsiella pneumoniae**	*Soares 2011* [40]	Labial and buccal mucosa		20								
**Escherichia coli**	*Soares 2011* [40]	Labial and buccal mucosa		0			8,3					
**Stenotrophomonas maltophilia**	*Soares 2011* [40]	Labial and buccal mucosa		0			8,3					
**Candida albicans**	*Soares 2011* [40]	Labial and buccal mucosa		60			33,3					
	*Mendoca 2012* [36]	Oral swab								25,4		14,9
**EBV**	*De Oliveira 2019* [32]	Cheek buccal mucosa from molars to incisors bilaterally				22						
**HSV**	*Mendoca 2012* [36]	Oral swab								14,3		6,3
**Fusobacterium nucleatum**	*Sixou 1998* [39]	Supragingival plaque of the last three teeth of the upper right quadrant and the last three teeth of the lower left						19	19	19	19	
	*Ye 2013* [41]	Lower lip and on the bucca	100		100							
**Viridans streptococchi**	*Sixou 1998* [39]	Supragingival plaque of the last three teeth of the upper right quadrant and the last three teeth of the lower left						50	44	75	63	
**Capnocytophaga**	*Sixou 1998* [39]	Supragingival plaque of the last three teeth of the upper right quadrant and the last three teeth of the lower left						81	81	50	56	
	*Ye 2013* [41]	Lower lip and on the bucca	100		0							
**Actinomyces odontolyticus**	*Sixou 1998* [39]	Supragingival plaque of the last three teeth of the upper right quadrant and the last three teeth of the lower left						38	31	44	31	

*D* Day, *Dx* Day of sampling either not specified or irregular

## Discussion

Oral mucositis is a common consequence of anti-neoplastic therapy in pediatric patients and a major issue due to its relevant impact on the quality of life of highly frail patients. Since the etiopathological mechanisms related to mucositis are presently not fully understood, every effort should be made to investigate all aspects and factors involved in its development.

The present review examined the literature to assess the available evidence on the role of oral microbiota in this regard. With the purpose of better analyzing the different agents, related to both different oncological diseases and therapies, involved in the complex process of oral mucositis development, only studies investigating patients undergoing chemotherapy were included, excluding other therapies such as radiotherapy or hematopoietic stem cell transplantation. Such a choice was made to point out the specific alteration of the microbiota of patients treated with antineoplastic drugs and its potential contribution to oral mucositis development.

Most of the analysed studies are not comparable due to discrepancies in design, times of observation and outcomes along with being at high risk of bias. This led to a high methodological heterogeneity of the included studies. Moreover, a mainly qualitative reporting of data of the included studies didn’t allow to perform a meta-analysis of results.

The mean prevalence of mucositis among the included studies was 57,6%, which is within the prevalence range defined in the scientific literature [18]. This indicates that more than half of pediatric patients undergoing cancer therapy with chemotherapy drugs develop mucositis.

The microbiological findings of the review will be discussed keeping the distinction made in the Results section, namely separating data obtained from studies either collecting samples directly from lesions or making a diagnosis of infection based on clinical aspects of lesions and studies collecting microbiological samples from standardized sites of the mouth.

In the first category, seven studies reported microbiological data obtained by direct sampling of sites showing signs of injury or clinically observing them. The mean frequency of Candida infection was 41% among the studies, with percentages of 52,3% and 61,7% in the works by Anirudhan et al. (2008) and Olczak-Kowalczyk et al. (2012) respectively which performed a proper microbiological test [29, 37].

Three studies reported the prevalence of oral lesions related to HSV infection, generally limited to a range between 3 and 9,7%.

It can be deduced from such results that in the development of oral lesions Candida infections frequently have a relevant role, while HSV plays a minor role.

Anirudhan et al. (2008) and Olczak-Kowalczyk et al. (2012) sampled oral lesions and performed broad spectrum microbiological analyses [29, 37]. Both studies observed a relatively frequent isolation of specific species, such as coagulase-negative *Staphylococci* and *S. viridans.* These data should be taken into consideration when designing prophylactic and therapeutic strategies to treat chemotherapy-induced mucositis.

In the second category, two studies evaluated the overall composition of the flora and its shift between the time of diagnosis of the neoplasm and the various stages of chemotherapy administration.

In the study by Sixou, et al., (1998) it was found that healthy patients, compared to oncologic patients undergoing chemotherapy, always had a greater complexity of microbial flora at days 0, 7, 14 and 21 of chemotherapy treatment [39]. Oncologic patients, however, have an unremarkable variation in microbiological complexity during therapy.

In the study by Ye et al. (2013), on the other hand, the UniFrac distance is greater in patients who have developed mucositis than in those who have not [41].

Even if results from these studies are not fully comparable, as the former compares oncologic patients with healthy patients and in the latter the groups were made of patients receiving chemotherapy who developed mucositis and patients who did not, findings from the studies seem to go in different directions. In addition to the difference in the study population of the two studies, there is a methodological distinction that can explain the apparent discrepancy in the results. In the study by Sixou et al. (1998) samples were cultured, according to the technology available, and mainly dental plaque-associated microorganisms (such as Porphyromonas spp., black-pigmented pre-votellae, Capnocytophaga sp., Fusobacterium nucleatum) were monitored. On the other side, Ye et al. (2013), thanks to the development of genomic-based methods of analysis such as PCR, performed a genetic testing that allowed for a wider identification of microorganisms isolated from the microbiological samples. Further studies are needed to clarify the general changes in the flora in the reported clinical situations.

With regard to the variation in the percentage of specific bacterial species, both reported articles evaluated the total anaerobic vital count of Capnocytophaga spp. in the study groups.

In Sixou’s study, the percentage of Capnocytophaga spp. was higher in healthy patients than in sick patients, and in sick patients there was no significant change during chemotherapy treatment [39].

In Ye’s study, on the other hand, there was a higher concentration of the bacterium in patients who developed mucositis than in those who did not [41]. Thus, Capnocytophaga spp. are found at higher concentrations in healthy patients according to Sixou et al., and in patients undergoing chemotherapy with the onset of mucositis according to Ye et al. [39, 41].

Regarding the prevalence of different microorganisms in oral sites without lesions, the observed frequencies exhibit heterogeneity. Coagulase-negative Staphylococci are significantly more represented in patients with chemotherapy-induced mucositis compared to those without mucositis. This finding aligns with previous investigations wherein direct sampling from lesions also identified coagulase-negative Staphylococci as some of the predominant microorganisms.

During chemotherapy treatment, Candida albicans manifests a frequency of 60% in patients with mucositis, as evidenced in the study conducted by Soares (2011), which concurs with the findings reported by Olczak-kowalczyk (2012) utilizing direct sampling from lesions. Interestingly, independent of the presence of mucositis, Mendoca’s study (2012) indicated a potential decline in the prevalence of Candida during chemotherapy treatment [36, 37, 40].

In summary, these observations underscore the variable prevalence of oral microorganisms in patients with chemotherapy-induced mucositis, with coagulase-negative Staphylococci exhibiting heightened prevalence and Candida albicans demonstrating increased frequency, albeit with a potential decrease during chemotherapy treatment, as suggested by Mendoca’s investigation [36].

In accordance with these findings, the systematic review with meta-analysis conducted by de Faria Gabriel et al. (2022) identified Candida spp. as risk factors for the development of mucositis in pediatric oncology patients. Furthermore, the review conducted by J. Napeñas et al. (2007) reports changes in the oral microbiome in studies involving children, specifically highlighting Gram-positive bacteria such as Streptococci and coagulase-negative Staphylococci [21, 22].

Main limitation of the study lies in the fact that the studies included in this systematic review exhibited substantial heterogeneity across various critical parameters, including chemotherapy regimens, administration of antimicrobials during chemotherapy, sampling sites, collection methodologies, collection timepoints, cultured microorganisms, mucositis assessment tools, and specific qualitative and quantitative endpoints. These divergences have posed challenges in establishing a definitive association between mucositis and alterations in the qualitative and/or quantitative composition of oral microbiota.

Same heterogeneity has been reported in J. Napeñas et al.’s (2007) review, which underscores the imperative of implementing standardized protocols within studies to attain greater coherence and consistency in research outcomes [22].

Another possible limitation of this study is the role of specific preventive/treatment protocols, wherever performed in the included studies, as possible confounding factors for microbiological outcomes. However, to date, there are no specific guidelines on prevention and/or treatment of mucositis, and a possible relationship between the drug/molecule used and microbiological variations hasn’t been clearly recorded. According to that, authors decided to not exclude all that studies that performed preventive and/or treatment protocols that could have had an impact on oral microbiota.

With all the declared limitations, this systematic review can contribute to better understand the role of the oral microbiota in the onset and progression of oral mucositis in pediatric oncology patients. Further studies are needed to acknowledge the main components involved in the change of the homeostasis of the oral microbiota, in order to design clinical protocols to prevent and treat oral mucositis, or at least, to provide pediatric patients the required assistance.

## Conclusion

The role of the oral microbiota, and the changes it undergoes during and after antineoplastic therapy, in the aetiopathogenesis of oral mucositis in the paediatric oncological population is a topic of great clinical interest, although concordant results are difficult to identify in the scientific literature produced to date.

The present systematic review showed an average prevalence of mucositis occurrence in the included studies of 57,6% of patients.

Some microbiological agents, such as Candida albicans, coagulase-negative Staphylococci and Streptococcus viridans were more frequently detected on lesion sites, whereas viruses such as herpes simplex did not seem to have the same degree of occurrence.

The shift in the flora’s relative abundance of species, does not provide clear guidelines on how to interpret the data currently available.

Further studies are necessary to implement knowledge on the subject to ensure a more effective diagnostic, prophylactic and therapeutic pathway. Thus, to manage a complication such as mucositis that significantly worsens the quality of life of a patient population already considered extremely fragile.

It could be useful, in order to improve the knowledge of oral microbiota in the development of oral mucositis in pediatric oncology patients a standardization of data collection and comparison of the same data, collected from healthy pediatric populations, oncologic children before the treatment and at the various times since the beginning of therapies. Once the role of the oral microbiota has been fully understood, future studies should focus on the identification of molecules or microorganisms (i.e. probiotics) aimed at re-establish the oral microbiota homeostasis, in order to prevent or manage oral mucositis.

## Data Availability

The datasets used and/or analysed during the current study available from the corresponding author on reasonable request.
